# Identification of a novel synthetic lethality of combined inhibition of hedgehog and PI3K signaling in rhabdomyosarcoma

**DOI:** 10.18632/oncotarget.2726

**Published:** 2015-03-06

**Authors:** Ulrike Graab, Heidi Hahn, Simone Fulda

**Affiliations:** ^1^ Institute for Experimental Cancer Research in Pediatrics, Goethe-University, Frankfurt, Germany; ^2^ Institute of Human Genetics, University Medical Center, Goettingen, Germany; ^3^ German Cancer Consortium (DKTK), Heidelberg, Germany; ^4^ German Cancer Research Center (DKFZ), Heidelberg, Germany

**Keywords:** apoptosis, hedgehog, PI3K, rhabdomyosarcoma

## Abstract

We previously reported that aberrant HH pathway activation confers a poor prognosis in rhabdomyosarcoma (RMS). Searching for new treatment strategies we therefore targeted HH signaling. Here, we identify a novel synthetic lethality of concomitant inhibition of HH and PI3K/AKT/mTOR pathways in RMS by GLI1/2 inhibitor GANT61 and PI3K/mTOR inhibitor PI103. Synergistic drug interaction is confirmed by calculation of combination index (CI < 0.2). Similarly, genetic silencing of GLI1/2 significantly increases PI103-induced apoptosis. GANT61 and PI103 also synergize to induce apoptosis in cultured primary RMS cells emphasizing the clinical relevance of this combination. Importantly, GANT61/PI103 cotreatment suppresses clonogenic survival, three-dimensional sphere formation and tumor growth in an *in vivo* model of RMS. Mechanistic studies reveal that GANT61 and PI103 cooperate to trigger caspase-dependent apoptosis via the mitochondrial pathway, as demonstrated by several lines of evidence. First, GANT61/PI103 cotreatment increases mRNA and protein expression of NOXA and BMF, which is required for apoptosis, since knockdown of NOXA or BMF significantly reduces GANT61/PI103-induced apoptosis. Second, GANT61/PI103 cotreatment triggers BAK/BAX activation, which contributes to GANT61/PI103-mediated apoptosis, since knockdown of BAK provides protection. Third, ectopic expression of BCL-2 or non-degradable phospho-mutant MCL-1 significantly rescue GANT61/PI103-triggered apoptosis. Fourth, GANT61/PI103 cotreatment initiate activation of the caspase cascade via apoptosome-mediated cleavage of the initiator caspase-9, as indicated by changes in the cleavage pattern of caspases (e.g. accumulation of the caspase-9 p35 cleavage fragment) upon addition of the caspase inhibitor zVAD.fmk. Thus, combined GLI1/2 and PI3K/mTOR inhibition represents a promising novel approach for synergistic apoptosis induction and tumor growth reduction with implications for new treatment strategies in RMS.

## INTRODUCTION

RMS, the most common pediatric soft-tissue sarcoma, can be classified into two major subtypes, i.e. alveolar (ARMS) and embryonal (ERMS), according to the mutation status and histological features [1, 2]. One common mutation in RMS consists in the loss of heterozygosity (LOH) at chromosomal region 9p22, including the *patched homolog* (*PTCH*) locus [3]. *PTCH* mutations also give rise to the naevoid basal cell carcinoma syndrome (NBCCS), known as Gorlin syndrome, which is characterized by predispositions to develop basal cell carcinomas (BCC), medulloblastoma (MB) and RMS [4–6]. Mice heterozygous for *PTCH* develop many of the features characteristic for NBCCS, including RMS [5, 7]. PTCH is an essential component of the HH signaling pathway, which is activated in RMS i.e. via loss of chromosomal region 9p22 or by amplification of the 12q13–15 region including the *GLI1* gene [3].

Canonical HH signaling pathway is activated via binding of one of the ligands, e.g. sonic hedgehog (SHH), to the transmembrane receptor PTCH. This leads to the inactivation of PTCH and subsequently to the release of the second transmembrane receptor smoothened (SMO) [8]. SMO in turn modulates expression and/or post-translational processing of the three GLI transcription factors. GLI1 and GLI2 act mainly as transcriptional activators, while GLI3 functions as a transcriptional repressor [9]. The balance between GLI activator and repressor forms results in expression of HH target genes, including *GLI1* and *PTCH* [10]. Besides the canonical HH pathway, GLI proteins can also be activated in a non-canonical and SMO-independent manner via phosphorylation by PI3K/AKT [11, 12], mTOR/S6 [13], RAS [11, 14] or MAPK/ERK [15].

Crosstalk between HH and PI3K/AKT/mTOR signaling has been observed in different tumor entities. In esophageal cancer, mTOR/S6 kinase signaling was shown to phosphorylate GLI1, promoting its transcriptional activity and tumor growth [13, 16]. In breast cancer, PI3K/AKT signaling was reported to protect key elements of the HH signaling pathway including GLI1 from proteasomal degradation [17].

Programmed cell death is a fundamental cellular program that is critical for maintaining tissue homeostasis [18]. Two major apoptosis signaling pathways have been characterized, i.e. the extrinsic, receptor-mediated and the intrinsic, mitochondria-mediated pathway [19]. Mitochondrial apoptosis is controlled by various factors including BCL-2 family proteins, which comprise antiapoptotic proteins such as BCL-2, BCL-X_L_ and MCL-1 as well as proapoptotic proteins like BAK, BAX and BH3-only proteins (i.e. BID, BIM, BMF and NOXA) [20]. Activation of BAX and BAK, for example upon binding of BH3-only proteins, leads to mitochondrial outer membrane permeabilization and release of mitochondrial intermembrane space proteins such as cytochrome C that engages caspase-9 activation within the apoptosome complex [21].

Since we previously identified aberrant activation of the HH pathway as a new poor prognostic factor in RMS [22], we aimed at therapeutic targeting of this signaling cascade in the present study. In view of mounting evidence showing that canonical as well as non-canonical mechanisms can cause HH activation, we inhibited HH signaling at different levels alone and in combination with inhibition of HH-interacting pathways such as PI3K/AKT/mTOR signaling.

## RESULTS

### GANT61 and PI103 synergize to induce apoptosis in RMS cells

To investigate the role of the HH signaling pathway in RMS we initially evaluated expression levels of different HH components in a panel of ARMS and ERMS cell lines. HH pathway activation was documented by broad expression of *GLI1* and *GLI2*, whereas *GLI3* and *PTCH* were differentially regulated and SHH expression was not detectable in most cell lines (Fig. S1A).

**Figure 1:**
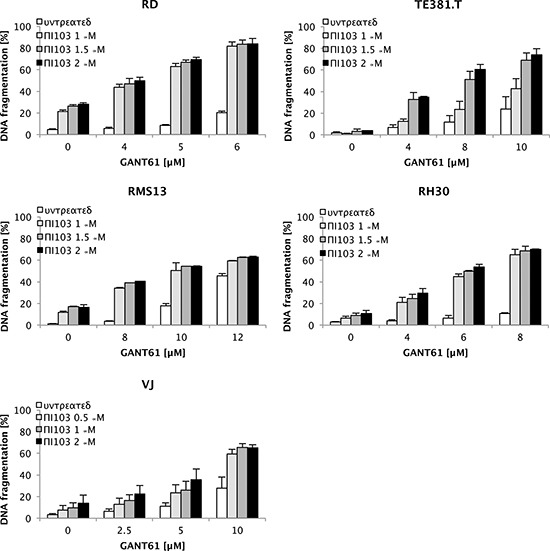
GANT61 and PI103 synergize to induce apoptosis in RMS cells RD, TE381.T, RMS13, RH30 and VJ cells were treated for 72 hours with indicated concentrations of PI103 and/or GANT61. Apoptosis was determined by DNA fragmentation of propidium iodide (PI)-stained nuclei using flow cytometry. Mean + S.D. of three independent experiments performed in triplicate are shown. Corresponding CI values are shown for all cell lines in Suppl. Table 1.

To represent the two major histological subtypes of RMS we selected the two alveolar RMS cell lines RMS13 and RH30, which contain an amplification of *GLI1* [23, 24] and the embryonal RMS cell lines RD and TE381.T. Importantly, we discovered that the GLI1/2 inhibitor GANT61 and the dual PI3K/mTOR inhibitor PI103 synergistically induced apoptosis in all RMS cell lines (Fig. 1A). Calculation of combination index (CI) illustrates the synergism of this combination treatment (Suppl. Table S1). Parallel experiments showed that GANT61 and PI103 cooperated to suppress mRNA levels of HH target genes *GLI1, GLI2* and *PTCH* (Fig. S2A) and to reduce phosphorylation of key components of the PI3K/AKT/mTOR pathway such as AKT, S6 and 4E-BP1 (Fig. S2B). To ensure that this finding is not restricted to established cell lines, we used primary cultured RMS cells derived from a tumor sample. Similarly, GANT61 and PI103 synergized to trigger apoptosis in primary cultured RMS cells (Fig. 1A), underlining the clinical relevance of this combination. By comparison, treatment with the SMO inhibitor GDC-0449 at micromolar concentrations exerted little effects on cell viability and HH target gene expression (Fig. S1B) and failed to cooperate with PI103 to induce apoptosis in RMS cells (Fig. S1C).

In addition to pharmacological inhibition of GLI1/2 by GANT61, we also tested a genetic approach by concomitant knockdown of GLI1/2 via siRNA. Similarly, GLI1/2 silencing significantly increased PI103-induced apoptosis (Fig. S3A). In addition to PI103 the dual PI3K/mTOR inhibitor BEZ235, the PI3K inhibitor GDC-0941 and the mTOR inhibitors RAD001 and AZD8055 all significantly increased GANT61-induced apoptosis (Fig. S4). This indicates that inhibition of PI3K/AKT/mTOR signaling at different levels interacts with HH pathway inhibition to trigger apoptosis in RMS cells. Together, this set of experiments shows that combined inhibition of GLI1/2 and PI3K/mTOR synergizes to induce apoptosis in RMS.

### GANT61/PI103 cotreatment cooperates to trigger caspase cleavage and caspase-dependent apoptosis

**Figure 2:**
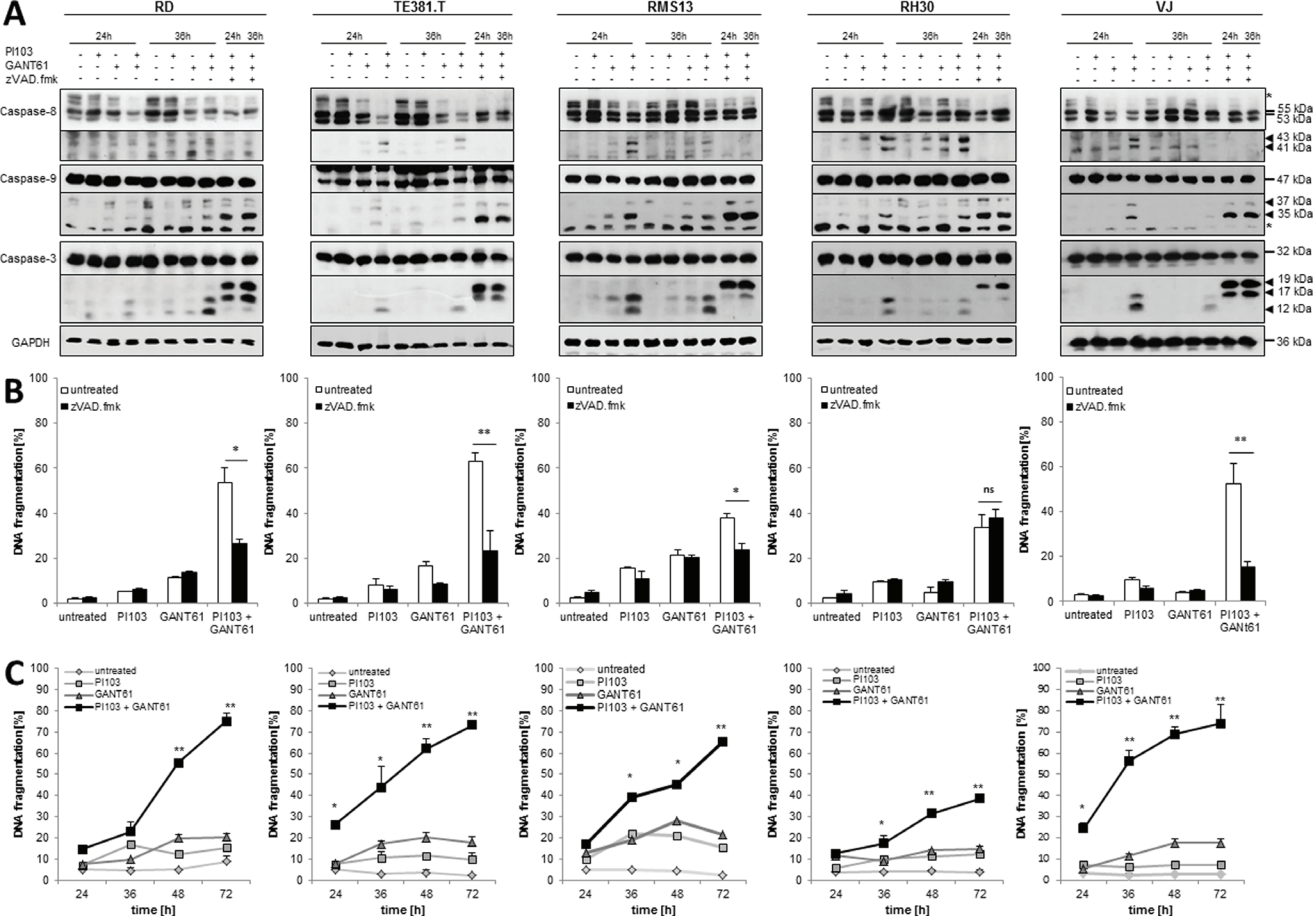
GANT61/PI103 cotreatment cooperates to trigger caspase cleavage and caspase-dependent apoptosis RD, TE381.T, RMS13, RH30 and VJ cells were treated for indicated times **(A, C)** or for 48 hours (B) with 1 μM PI103 and/or GANT61 (RD 6 μM; TE381.T 8 μM; RMS13 10 μM; RH30 8 μM; VJ 8 μM) in the presence or absence of 50 μM zVAD.fmk (A, B). In A, caspase activation was analyzed by Western blotting (asterisks indicate unspecific bands) and active cleavage fragments are indicated by arrow heads. **(B and C)** apoptosis was determined by DNA fragmentation of PI-stained nuclei using flow cytometry. Mean + S.D. of three independent experiments performed in triplicate (B, C) or representative blots (A) are shown; *p < 0.05; **p < 0.01.

To investigate the underlying molecular mechanisms of the synergistic interaction of GANT61 and PI103 we analyzed activation of caspases involved in intrinsic and extrinsic apoptosis signaling. GANT61 and PI103 cooperated to trigger cleavage of caspase-9 into p37 and p35 fragments, caspase-3 into p17 and p12 active cleavage fragments and caspase-8 into p43 and p41 fragments (Fig. 2A). To test whether caspase activity is necessary for apoptosis we used the broad-range caspase inhibitor N-benzyloxycarbonyl-Val-Ala-Asp-fluoromethylketone (zVAD.fmk). Addition of zVAD.fmk significantly reduced GANT61/PI103-induced apoptosis in all cell lines except RH30 cells (Fig. 2B). Interestingly, zVAD.fmk treatment changed the cleavage pattern of caspase-9 and -3 with accumulation of caspase-9 p35 and caspase-3 p19 cleavage fragments, whereas caspase-8 cleavage was completely blocked (Fig. 2A). These findings point to apoptosome-mediated cleavage of caspase-9 via induced proximity independently of caspase activity and subsequent initial cleavage of caspase-3 by caspase-9, while autoproteolytic processing of caspase-3 is blocked by zVAD.fmk. Kinetic analysis revealed that GANT61/PI103-induced apoptosis started around 24 hours in TE381.T and VJ cells and slightly later in RD, RMS13 and RH30 cells (Fig. 2C). Taken together, these findings indicate that GANT61/PI103 cotreatment cooperates to induce caspase activation and caspase-dependent apoptosis.

### GANT61/PI103 cotreatment increases NOXA and BMF expression

**Figure 3:**
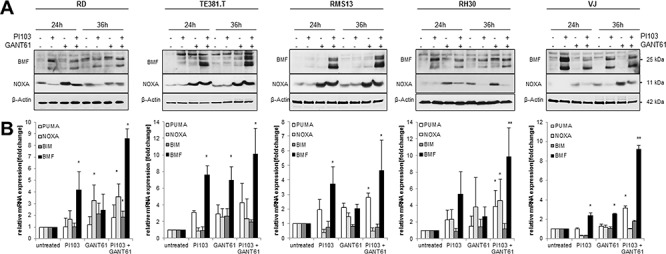
GANT61/PI103 cotreatment increases NOXA and BMF expression RD, TE381.T, RMS13, RH30 and VJ cells were treated with 1 μM PI103 and/or GANT61 (RD 6 μM; TE381.T 8 μM; RMS13 10 μM; RH30 8 μM; VJ 8 μM) for indicated times **(A)** or 24 hours **(B)** A, protein expression of BMF and NOXA was analyzed by Western blotting. B, mRNA levels of pro-apoptotic BCL-2 proteins were determined by qRT-PCR. Mean + S.D. of three independent experiments performed in triplicate (B) or representative blots (A) are shown; *p < 0.05; **p < 0.01 comparing treated to untreated cells.

Since the observed cleavage pattern of caspases points to engagement of the mitochondrial apoptotic pathway by GANT61/PI103 cotreatment, we analyzed the effects of GANT61 and PI103 on expression levels of pro- and antiapoptotic BCL-2 family proteins, which play an important role in regulating mitochondrial apoptosis. Interestingly, treatment with GANT61 alone or in combination with PI103 led to upregulation of the proapoptotic protein NOXA (Fig. 3A), which was accompanied by upregulation of NOXA mRNA by GANT61/PI103 cotreatment in RD and RH30 cells (Fig. 3B). In addition, treatment with PI103 alone or in combination with GANT61 caused a substantial upregulation of BMF mRNA and protein levels (Fig. 3A, 3B). Furthermore, GANT61/PI103 cotreatment increased expression of BIM and reduced MCL-1 protein levels in RH30 cells, whereas it had little effects on expression of BCL-2, BCL-X_L_, BAX and BAK (Fig. S5). This suggests that GANT61/PI103 cotreatment shifts the ratio of pro- and antiapoptotic BCL-2 proteins towards apoptosis.

### NOXA and BMF are required for GANT61/PI103-induced apoptosis

**Figure 4:**
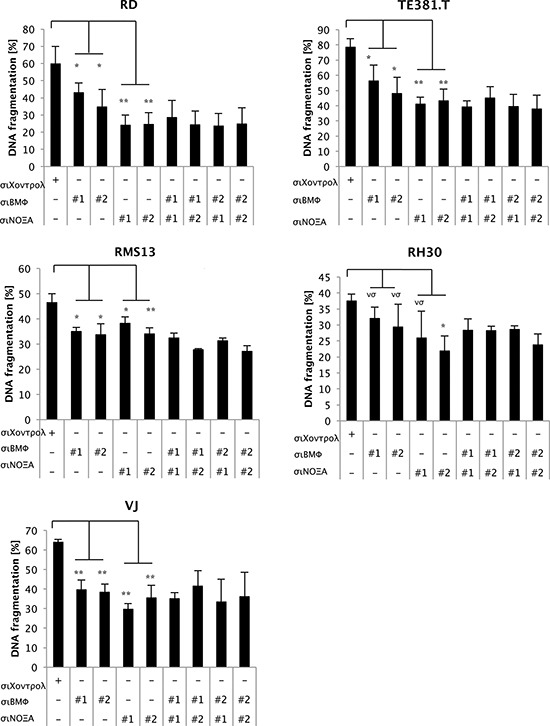
NOXA and BMF are required for GANT61/PI103-induced apoptosis RD, TE381.T, RMS13, RH30 and VJ cells were transfected with non-silencing siRNA (siControl) or siRNA targeting BMF and/or NOXA and treated for 48 hours with PI103 (1 μM) and GANT61 (RD 6 μM; TE381.T 8 μM; RMS13 10 μM; RH30 8 μM; VJ 8 μM). Apoptosis was determined by DNA fragmentation of PI-stained nuclei using flow cytometry. Mean + S.D. of at least three independent experiments performed in triplicate are shown; *p < 0.05; **p < 0.01. Statistic analysis comparing combined knockdown to single knockdown is shown in Suppl. Table 2.

To investigate the functional involvement of NOXA and BMF in apoptosis induction we knocked down these proteins by siRNA (Fig. S6). NOXA silencing significantly reduced GANT61/PI103-induced apoptosis in all cell lines and BMF knockdown significantly decreased apoptosis in all but RH30 cells (Fig. 4A). Simultaneous knockdown of both NOXA and BMF led to a further significant reduction of GANT61/PI103-induced apoptosis in RMS13 cells (Suppl. Table S2). These findings demonstrate that NOXA and BMF contribute to GANT61/PI103-induced apoptosis.

### GANT61/PI103-mediated BAX/BAK activation is required for apoptosis

**Figure 5:**
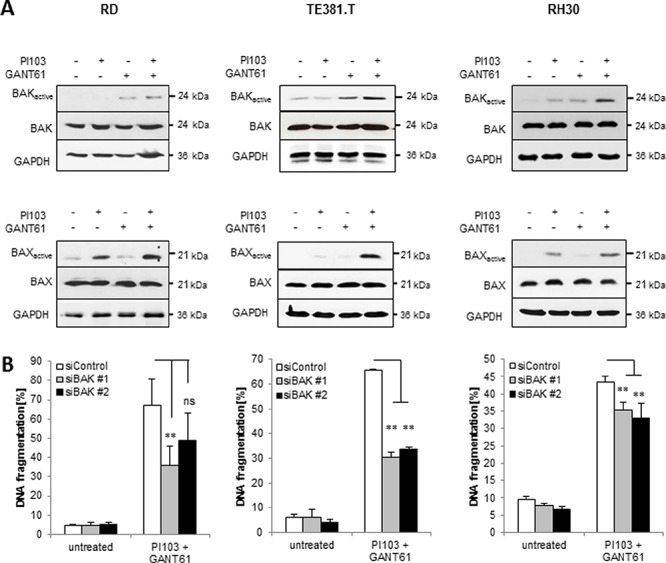
GANT61/PI103-mediated BAX/BAK activation is required for apoptosis **(A)** RD, TE381.T and RH30 cells were treated with 1 μM PI103 and/or GANT61 (RD 6 μM; TE381.T 8 μM; RH30 8 μM) and activation of BAK and BAX was determined after 24 hours by immunoprecipitation using active conformation-specific antibodies; representative blots are shown. **(B)** cells were transfected with non-silencing siRNA (siControl) or siRNA targeting BAK, treated with 1 μM PI103 and GANT61 (RD 6 μM; TE381.T 8 μM; RH30 8 μM) for 48 hours and apoptosis was determined by DNA fragmentation of PI-stained nuclei using flow cytometry. Mean + S.D. of three independent experiments performed in triplicate are shown; *p < 0.05; **p < 0.01; ns: not significant.

Next, we investigated the question whether the observed upregulation of NOXA and BMF causes activation of BAX and BAK. To this end, we immunoprecipitated BAK and BAX by conformation-specific antibodies, which specifically bind to their activated forms. Indeed, treatment with GANT61 and/or PI103 resulted in activation of BAX and/or BAK (Fig. 5A). Importantly, genetic knockdown of BAK by siRNA (Fig. S7) significantly reduced GANT61/PI103-induced apoptosis (Fig. 5B). These experiments demonstrate that GANT61/PI103-mediated BAX/BAK activation is required for apoptosis.

### Overexpression of BCL-2 or phospho-mutant MCL-1 rescues GANT61/PI103-induced apoptosis

**Figure 6:**
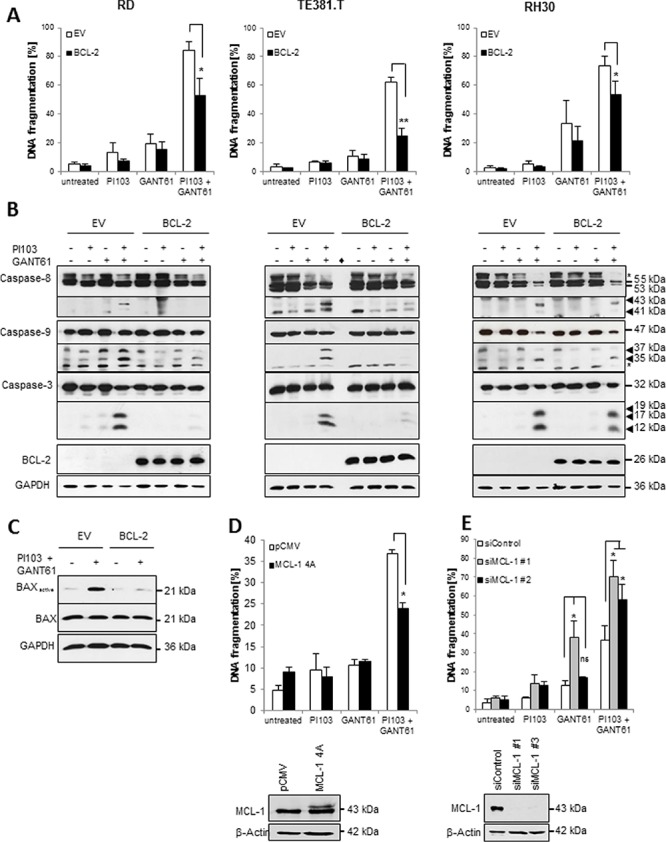
Overexpression of BCL-2 and phospho-mutant MCL-1 rescue GANT61/PI103-induced apoptosis **(A–C)**, RD, TE381.T and RH30 cells were transfected with a murine BCL-2 construct or empty vector and treated with 1 μM PI103 and GANT61 (RD 6 μM; TE381.T 8 μM; RH30 8 μM). A, apoptosis was determined after 48 hours by DNA fragmentation of PI-stained nuclei using flow cytometry. B, caspase activation was determined by Western blotting after 24 hours; arrow heads indicate cleavage fragments; asterisks indicate unspecific bands; diamond indicates empty lane. C, BAX activation was determined after 24 hours by immunoprecipitation using active conformation-specific antibodies. **(D)** RD cells were transfected with a non-degradable phospho-mutant MCL-1 construct or empty vector, treated with 1 μM PI103 and 6 μM GANT61 and apoptosis was determined after 48 hours by DNA fragmentation of PI-stained nuclei using flow cytometry (upper panel). Overexpression of the MCL-1 construct was verified by Western blotting (lower panel). **(E)** RD cells were transfected with non-silencing siRNA (siControl) or two different siRNAs targeting MCL-1 and apoptosis was determined after 48 hours by DNA fragmentation of PI-stained nuclei using flow cytometry (upper panel). MCL-1 knockdown was verified by Western blotting (lower panel). Mean + S.D. of three independent experiments performed in triplicate (A, D, E) or representative blots (B–E) are shown; *p < 0.05; **p < 0.01; ns: not significant.

To confirm the requirement of the mitochondrial pathway for GANT61/PI103-induced apoptosis, we overexpressed BCL-2 or a non-degradable phospho-deficient MCL-1 mutant [25]. Overexpression of BCL-2 significantly decreased GANT61/PI103-induced apoptosis, attenuated cleavage of caspase-3, -8 and -9 and prevented GANT61/PI103-induced activation of BAX (Fig. 6A–6C). Also, ectopic expression of MCL-1 mutant significantly decreased GANT61/PI103-induced apoptosis (Fig. 6D). Vice versa, knockdown of MCL-1 via siRNA significantly increased GANT61/PI103-induced apoptosis (Fig. 6E). These findings confirm that signaling via an intact mitochondrial pathway is required for GANT61/PI103-induced apoptosis.

### GANT61 and PI103 cooperate to suppress clonogenic survival, sphere formation and tumor growth *in vivo*

**Figure 7:**
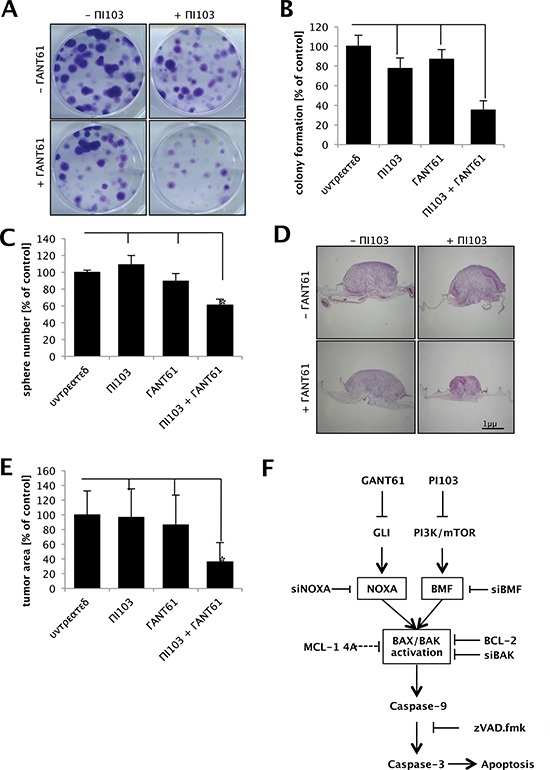
GANT61 and PI103 cooperate to suppress clonogenic survival, 3D sphere formation and tumor growth *in vivo* **(A and B)** RD cells were treated for 18 hours with 1 μM PI103 and/or 6 μM GANT61 and clonogenic survival was assessed by colony formation assay at day 10. Representative images are shown (A); the number of colonies was counted after crystal violet staining and is expressed as percentage of untreated controls (B) **(C)** RD cells were treated for 24 hours with 1 μM PI103 and/or 6 μM and spheres were counted after 15 days. **(D and E)**, RD cells were seeded on the CAM of fertilized chicken eggs, treated with 2 μM PI103 and/or 20 μM GANT61 for three days and tumor growth was analyzed using H/E-stained paraffin sections of the CAM. Representative pictures of at least 15 tumors (D) and quantification of tumor area (E) are shown. Mean + S.D. of three independent experiments performed at least in triplicate are shown (B, C, E); *p < 0.05; **p < 0.01. **(F)** Scheme of the proposed mechanism of GANT61/PI103 induced mitochondrial apoptosis. GANT61 and PI103 treatment results in NOXA and BMF upregulation and subsequently BAX and BAK activation. BAX/BAK activation leads to cleavage of caspase-9 and activation of caspase-3 as execution pathway of apoptosis. GANT61/PI103-induced apoptosis is inhibited by overexpression of BCL-2 or phospho-mutant MCL-1, by knockdown of NOXA, BMF or BAK or by caspase inhibitor zVAD.fmk.

Next, we investigated the effects of GANT61/PI103 co-treatment on clonogenic survival and three-dimensional (3D) tumor growth. GANT61 and PI103 cooperated to significantly reduce long-term clonogenic survival of RMS cells compared to either agent alone or to control (Fig. 7A, 7B). Also, GANT61/PI103 co-treatment significantly decreased sphere formation in a 3D culture model (Fig. 7C). Finally, we tested the *in vivo* antitumor activity of GANT61/PI103 co-treatment using the chicken CAM model, an established model for tumor growth [26]. To this end, RMS cells were seeded on the CAM of chicken embryos and allowed to form tumors followed by treatment with GANT61 and/or PI103 for three days. Importantly, GANT61 and PI103 acted in concert to reduce tumor growth (Fig. 7D, 7E). These experiments show that GANT61/PI103 co-treatment suppresses clonogenic survival, sphere formation and RMS growth *in vivo*.

## DISCUSSION

Searching for new treatment strategies in RMS, we focused on the HH signaling pathway, since we previously reported that aberrant activation of this pathway confers a poor prognosis in RMS [22]. There is accumulating evidence showing that the HH pathway is not only activated via canonical signaling, but also in a non-canonical manner, which may lead to resistance against SMO inhibitors [27]. Therefore, we targeted HH signaling at different levels alone or in combination with inhibition of HH-interacting pathways.

In this study, we identify a synthetic lethal interaction of concomitant inhibition of HH and PI3K/AKT/mTOR pathways in RMS. The combination of the GLI1/2 inhibitor GANT61 and PI3K/mTOR inhibitor PI103 is highly synergistic at subtoxic concentrations of both inhibitors as underscored by calculation of CI values (CI < 0.2). Similarly, genetic silencing of GLI1 and GLI2 cooperates with PI103 to induce apoptosis. The clinical relevance of this combination is emphasized by experiments using a patient-derived primary tumor sample, which similarly demonstrates the synergistic interaction of GANT61/PI103 cotreatment. The potency of this combination therapy is underlined by our data showing that GANT61/PI103 cotreatment reduces clonogenic survival, suppresses 3D rhabdosphere formation and, most importantly, inhibits tumor growth in an *in vivo* model of RMS. Based on these findings, we conclude that combined GLI1/2 and PI3K/mTOR inhibition is a promising novel therapeutic approach for synergistic apoptosis induction and tumor growth reduction in RMS.

Mechanistic studies revealed that the synergistic interaction of GANT61/PI103 cotreatment is mediated by the mitochondrial pathway of apoptosis via upregulation of the proapoptotic proteins NOXA and BMF, followed by activation of BAX and BAK and subsequent cleavage of caspases, as demonstrated by several lines of evidence (Fig. 7F).

First, single treatment with either inhibitor alone leads to upregulation of different proapoptotic BH3-only proteins, which is further increased after combination treatment. PI103 single treatment upregulates BMF, while GANT61 single treatment increases NOXA levels. PI3K/AKT/mTOR inhibition has been shown to repress CAP-dependent protein translation resulting in a shift towards internal ribosome entry site (IRES)-mediated translation and upregulation of BMF [28]. Also, it is interesting to note that arsenic trioxide, which can decrease GLI1 and GLI2 levels [29, 30], has been reported to increase NOXA mRNA and protein expression [31]. This emphasizes that HH inhibition can lead to upregulation of NOXA. Our findings showing that knockdown of NOXA or BMF attenuates GANT61/PI103-induced apoptosis highlight the functional relevance and non-redundant functions of these BH3-only proteins during GANT61/PI103-mediated apoptosis. The notion that signaling via the mitochondrial pathway is required for the synergistic induction of apoptosis is underscored by rescue experiments showing that inhibition of the mitochondrial pathway by overexpression of BCL-2, by ectopic expression of a non-degradable MCL-1 mutant or by BAK silencing provides protection against GANT61/PI103-induced apoptosis.

Second, GANT61 and PI103 cooperate to trigger caspase activation via the mitochondrial pathway of apoptosis, as indicated by changes in the cleavage pattern of caspases upon addition of the broad-range caspase inhibitor zVAD.fmk. Accordingly, accumulation of the caspase-9 p35 cleavage fragment upon addition of zVAD.fmk to GANT61/PI103-treated cells is consistent with cleavage of the initiator caspase-9 via induced proximity within the apoptosome, an event that does not require caspase activity and therefore is not inhibited by zVAD.fmk [32]. By comparison, reduced generation of the p37 cleavage fragment of caspase-9 upon GANT61/PI103 co-treatment in the presence of zVAD.fmk is in line with the notion that caspase-3 cleaves caspase-9 into the 37-kDa fragment in a feedback amplification loop [33]. Accumulation of the 19-kDa fragment of caspase-3 in GANT61/PI103-treated cells in the presence of zVAD.fmk is consistent with increased caspase-9 activity, whereas subsequent autoproteolytic processing of caspase-3 into the 12- and 17-kDa fragments is inhibited by zVAD.fmk [34]. Slight activation of caspase-8 upon GANT61/PI103 cotreatment is likely caused by one of the effector caspases such as caspase-3, as it is blocked by zVAD.fmk. Caspase-8 may initiate a feedback amplification loop, for example by cleaving BID into tBID, which further enhances GANT61/PI103-induced apoptosis. Together, these mechanistic studies underscore that the mitochondrial pathway plays a critical role in mediating GANT61/PI103-induced apoptosis.

Canonical as well as non-canonical mechanisms can cause aberrant HH pathway activation in RMS. Inherited mutations in *PTCH* can give rise to RMS in both humans and mice, and human sporadic RMS samples were reported to harbor mutations in *PTCH* or *SUFU* as well as amplification of *GLI1* and *GLI2* loci [24, 35–40]. Furthermore, RMS often exhibit active PI3K/AKT/mTOR signaling [41] that contributes to non-canonical HH activation via phosphorylation of GLI1, which potentiates its transcriptional activity [13]. In line with non-canonical HH activation in RMS, our study reveals that concomitant inhibition of GLI and PI3K/mTOR signaling is required to induce apoptosis in RMS cells. Consistently, GANT61/PI103 cotreatment leads to profound and sustained suppression of both HH and PI3K/AKT/mTOR signaling compared to either treatment alone, highlighting the crosstalk of these pathways downstream of SMO. By comparison, we demonstrate that treatment with the SMO inhibitor GDC-0449 alone or in combination with PI3K/mTOR inhibition fails to induce apoptosis in RMS cells. As far as the RMS cell lines used in the present study are concerned, RH30 and RMS13 cells have been described to harbor *GLI1* amplification [23, 24]. Also, inhibition of GLI has previously been reported to be more efficient in RMS cell lines than inhibition of SMO by cyclopamine or by genetic silencing [42].

While tumors with documented HH activation caused by LOH of *PTCH* such as BCC have been described to respond to SMO inhibitors [43], there is mounting evidence showing that these inhibitors exhibit limited efficiency against tumors with non-canonical HH activation, e.g. due to mutations downstream of SMO, *GLI* amplification or activation of HH-interacting pathways such as the PI3K/AKT/mTOR cascade [13, 16, 44, 45]. In glioblastoma harboring PI3K/mTOR activation due to *PTEN* deficiency, combined inhibition using the SMO inhibitor LDE225 and the PI3K inhibitor BKM120 was necessary to reduce cell viability and inhibit tumor growth [12]. Likewise, cotreatment with the SMO inhibitor GDC-0449 and the mTOR inhibitor RAD001 suppressed tumor growth of esophageal cancer *in vivo* [13]. These studies highlight the need of concomitant inhibition of HH and PI3K/AKT/mTOR pathways in tumors with non-canonical HH pathway activation. This approach is currently under evaluation in clinical trials in pancreatic cancer (i.e. GDC-0449 and Rapamycin) and in advanced solid tumors (i.e. LDE225 and BKM120) [45].

Co-targeting of HH and PI3K/AKT/mTOR pathways not only provides a promising strategy to bypass primary resistance to SMO inhibitors due to non-canonical HH activation, but also to overcome secondary resistance to these inhibitors. The development of resistance has been documented upon monotherapy with SMO inhibitors both in humans and mice, e.g. due to acquired mutations in *SMO, GLI2* amplification or PI3K/AKT/mTOR activation [27, 46, 47]. For example in a mouse model of medulloblastoma, addition of PI3K inhibitor BKM120 or PI3K/mTOR inhibitor BEZ235 to the initial treatment with SMO inhibitor LDE225 markedly delayed the occurrence of resistance [47].

Our study has important implications for the development of HH pathway-targeted therapies in RMS. The identification of synthetic lethality by combined inhibition of HH signaling at the level of GLI and of PI3K/mTOR in the present study is relevant for RMS, since non-canonical mechanisms contribute to aberrant HH pathway activation. Beyond RMS, our results are also of broader relevance for other HH-driven cancer with non-canonical HH activation. One future challenge is the development of clinically applicable GLI-targeting drugs. Here, inhibition of bromo and extra C-terminal (BET) bromodomain proteins may open new perspectives, as the small-molecule BET bromodomain inhibitor JQ1 has recently been reported to block GLI transcriptional output downstream of SMO [48]. Taken together, HH pathway-targeted therapies may open new perspectives for more effective treatment options for RMS.

## METHODS

### Cell culture and chemicals

RMS cell lines were obtained from the American Type Culture Collection (Manassas, VA, USA) and maintained in RPMI 1640 or DMEM medium (Life Technologies., Eggenstein, Germany), supplemented with 10% fetal calf serum (FCS) (Biochrom, Berlin, Germany), 1 mM glutamine and 1% penicillin/streptomycin (Invitrogen, Karlsruhe, Germany). VJ cells were generated from a primary cultured RMS derived from a patient diagnozed with RMS (histologically confirmed alveolar rhabdomyosarcoma) and cultured in DMEM medium. zVAD.fmk was purchased from Bachem (Heidelberg, Germany). GDC-0449 [49] and GDC-0941 were kindly provided by Genetech Inc. (South San Francisco, CA, USA), PI3K/mTOR inhibitor PI103 [50] was purchased from Merck Millipore (Darmstadt, Germany) and GLI1/2 inhibitor GANT61 [51] from Sigma (Deisenhofen, Germany). BEZ235 and RAD001 were kindly provided by Novartis Institute for BioMedical Research (Oncology Basel, Novartis Pharma AG, Basel Switzerland). AZD8055 was obtained from Selleck Chemicals (Houston, Texas). All chemicals were purchased from Sigma unless indicated otherwise.

### Determination of apoptosis and cell viability

Apoptosis was determined by analysis of DNA fragmentation of propidium iodide (PI)-stained nuclei using flow cytometry (FACSCanto II, BD Biosciences, Heidelberg, Germany) as described previously [52]. Cell viability was assessed by 3-(4,5-dimethylthiazol-2-yl)- 2,5-diphenyltetrazolium bromide (MTT) assay according to the manufacturer's instructions (Roche Diagnostics, Mannheim, Germany).

### Determination of colony formation and sphere formation

For colony assay, cells were treated for 18 hours, re-seeded as single cells (200 cells/well) in six-well plates and cultured for additional 10 days before colonies were stained with crystal violet (Roth, Karlsruhe, Germany) and counted. For sphere formation, cells were treated for 24 hours and seeded as single cells in stem cell culture medium in ultra low attachment plates as previously described [53].

### Western blot analysis

Western blot analysis was performed as described previously [52] using the following antibodies: mouse anti-caspase-8, mouse anti-NOXA, rat anti-BMF (Alexis Biochemicals, Grünberg, Germany), mouse anti-AKT, mouse anti-BCL-2, mouse anti-BAX, rabbit anti-BAK (BD Bioscience), rabbit anti-caspase-3, mouse anti-caspase-9, rabbit anti-pAKT, rabbit anti-p4E-BP1, rabbit anti-4E-BP1, rabbit anti-pS6, mouse anti-S6, (Cell Signaling, Beverly, MA), rabbit anti-MCL-1 (Stressgene Bioreagents, Victoria, BC). Mouse anti-GAPDH (HyTest, Turku, Finland) or mouse anti-β-Actin (Sigma) were used as loading controls. Goat anti-mouse IgG, donkey anti-goat IgG, goat anti-rabbit IgG conjugated to horseradish peroxidase (Santa Cruz Biotechnology, Santa Cruz, CA)) and goat anti-mouse IgG1 or goat anti-mouse IgG2b (Southern Biotech, Birmingham, AL) conjugated to horseradish peroxidase were used as secondary antibodies. Enhanced chemiluminescence was used for detection (Amersham Bioscience, Freiburg, Germany). Representative blots of at least two independent experiments are shown.

### BAX/BAK activation

A total of 1000 μg protein was immunoprecipitated with mouse anti-BAX antibody (6A7, Sigma) or mouse anti-BAK (TC-100, Merck Millipore) and 5 μl Dynabeads Pan Mouse IgG (Dako, Hamburg, Germany). The precipitate was analyzed by Western blotting using the BAX NT antibody (Merck Millipore) or BAK antibody (BD, Bioscience).

### Statistical analysis

Statistical significance was assessed by Student's *t*-Test (two-tailed distribution, two-sample, unequal variance). Interaction between PI103 and GANT61 was analyzed by the Combination index (CI) method based on that described by [54] using CalcuSyn software (Biosoft, Cambridge, UK). CI < 0.9 indicates synergism, 0.9–1.1 additivity and > 1.1 antagonism.

### Transient RNA interference

For transient knockdown by siRNA, cells were reversely transfected with 10 nM SilencerSelect siRNA (Invitrogen), control siRNA (4390843) or targeting siRNAs (s8583 and s8585 for MCL-1; s40385 and s40386 for BMF, s10708 and s10709 for NOXA, s5814 for GLI1, s5817 for GLI2, s1880 and s1881 for BAK) using Lipofectamine RNAi Max (Invitrogen) and OptiMEM (Life Technologies).

### Transduction

For BCL-2 overexpression, cells were transduced with murine stem cell virus (pMSCV, Clontech, Mountain View, CA) vector containing mouse BCL-2 or empty vector using the packaging cell line 293T (BD Biosciences). Stable cell lines were selected with 10 μg/ml Blasticidin (Invitrogen). For phospho-mutant MCL-1, cells were transduced with a pCMV vector containing 4A (S64A/S121A/S159A/T163A) and empty vector by Genentech (South San Francisco) and were selected with Neomycin (Roth).

### CAM assay

CAM assay was done as described previously [26]. Briefly, 10^6^ cells were implanted on fertilized chicken eggs on day eight of incubation, treated with 2 μM PI103 and 20 μM GANT61 for three days, sampled with the surrounding CAM, fixed in 4% paraformaldehyde, paraffin embedded, cut in 5-mm sections and were analyzed by immunohistochemistry using 1:1 hematoxylin and 0.5% eosin (H/E). Images were digitally recorded using an AX70 microscope (Olympus, Center Valley, PA, USA) and tumor areas were analyzed by ImageJ digital imaging software (NIH, Bethesda, MA, USA).

### Quantitative real-time PCR

Total RNA was extracted using peqGOLD Total RNA kit from Peqlab Biotechnologie GmbH (Erlangen, Germany) according to the manufacturer's instructions. Total RNA (2 μg) was used to synthesize the corresponding cDNA using RevertAid H Minus First Strand cDNA Synthesis Kit (MBI Fermentas GmbH, St. Leon-Rot, Germany). To quantify gene expression levels, SYBRGreen based quantitative RT-qPCR was performed using the 7900HT fast real-time PCR system from Applied Biosystems (Darmstadt, Germany). Data were normalized on 28S-rRNA expression as reference gene. Primers are listed in Suppl. Table S3. Melting curves were plotted to verify the specificity of the amplified products. The relative expression of the target gene transcript and reference gene transcript was calculated as ΔΔc_t_. At least two independent experiments were performed for each gene.

## SUPPLEMENTARY FIGURES AND TABLES


