# Supplementary material for: Identification of a novel synthetic lethality of combined inhibition of hedgehog and PI3K signaling in rhabdomyosarcoma
Source: Oncotarget. 2015 Mar 6;6(11):8722–35. doi: 10.18632/oncotarget.2726 (PMC4496179; doi:10.18632/oncotarget.2726)
Supplement: Supplementary file 1 [file oncotarget-06-8722-s001.pdf]

SUPPLEMENTARY FIGURES AND TABLES

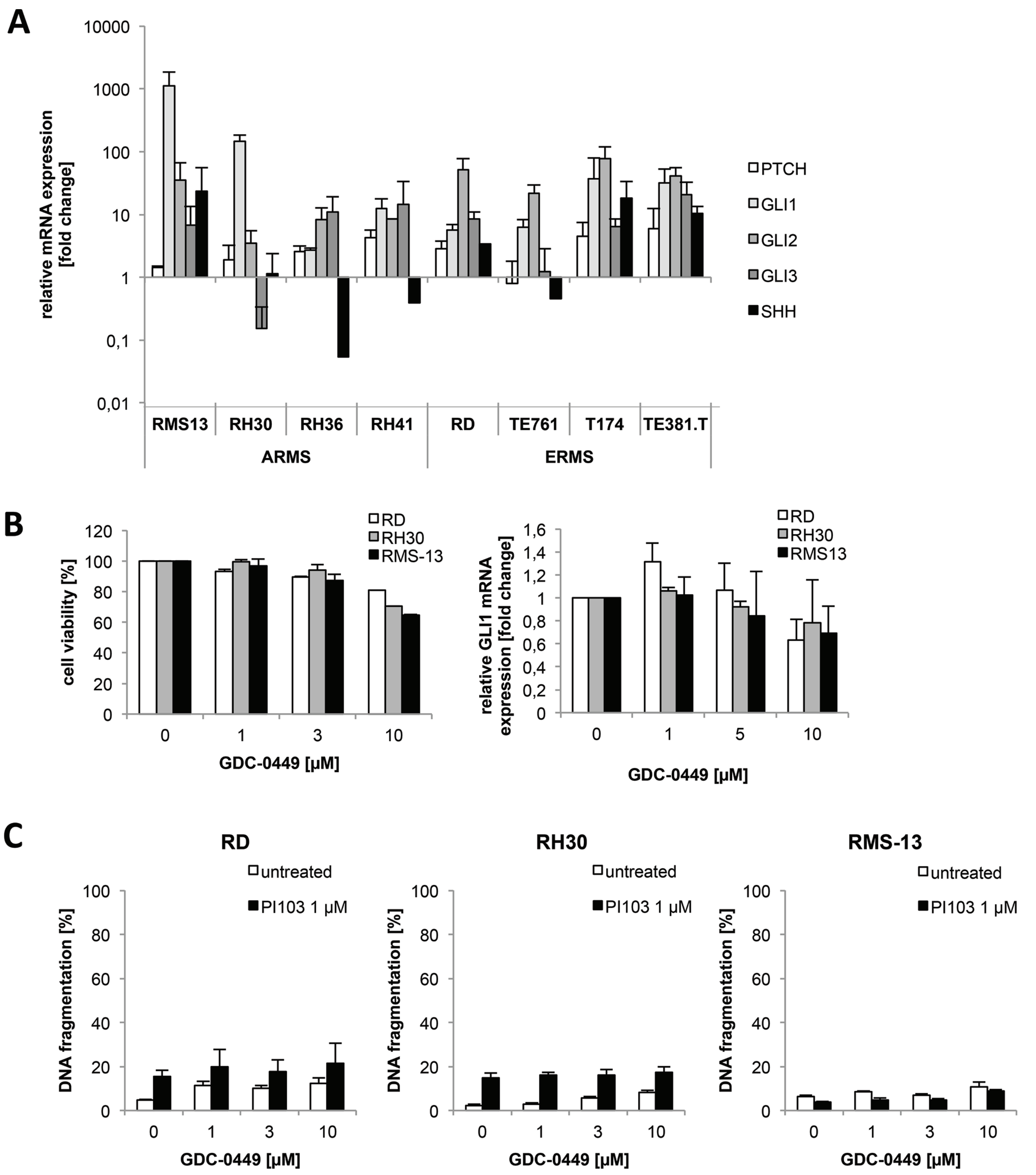

Supplementary Figure S1: Hedgehog expression in RMS cell lines and effects of GDC-0449. (A) mRNA levels of HH pathway components were determined by qRT-PCR in ARMS and ERMS cell lines and normalized to human skeletal muscle mRNA levels. **(B)** RD, RH30 and RMS13 cells were treated with indicated concentrations of GDC-0449 and cell viability was measured by MTT assay after 72 hours and is expressed as the percentage of untreated controls (left panel). GLI1 mRNA levels were determined by qRT-PCR after 48 hours (right panel). **(C)** RD, RH30 and RMS13 cells were treated with indicated concentrations of GDC-0449 and/or 1 µM PI103 for 72 hours and apoptosis was determined by DNA fragmentation of PI-stained nuclei using flow cytometry. Mean +/– S.D. of three independent experiments performed in triplicate are shown.

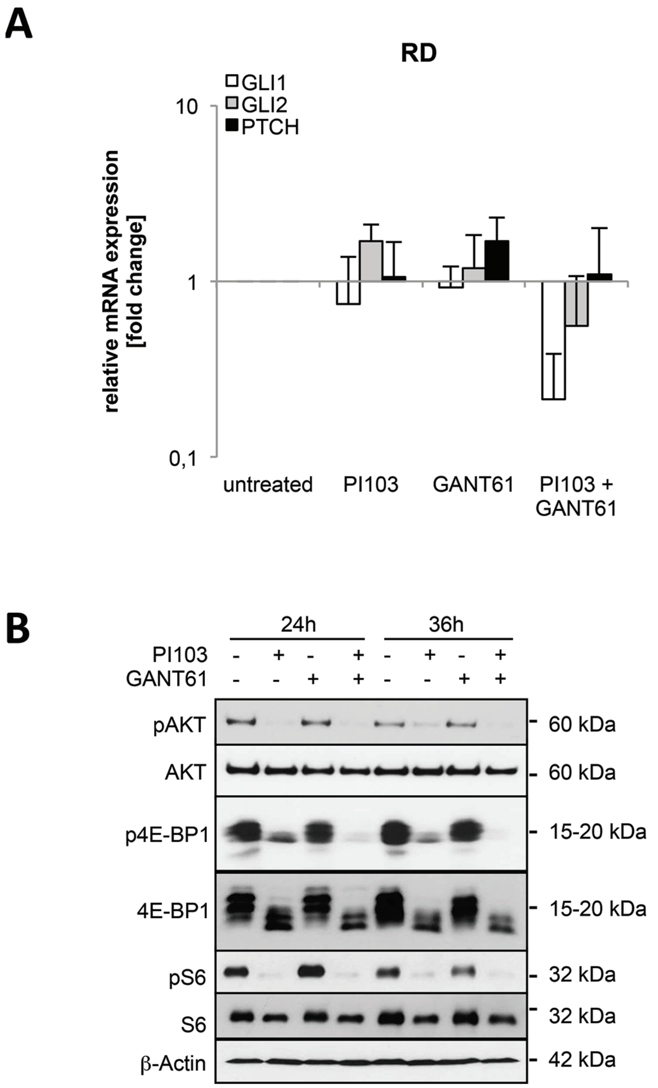

Supplementary Figure S2: GANT61/PI103 cotreatment in RMS cells. (A) RD and RH30 were treated with 1 µM PI103 and/or GANT61 (RD 6 µM; RH30 8 µM) for 24 hours, mRNA levels of GLI1, GLI2 and PTCH were determined by qRT-PCR and are normalized to untreated cells. **(B)** RD cells were treated for 24 and 36 hours with 1 µM PI103 and/or 6 µM GANT61 and protein levels were determined by Western blotting. Mean +/– S.D. of three independent experiments performed in triplicate (A) or representative blots (B) are shown.

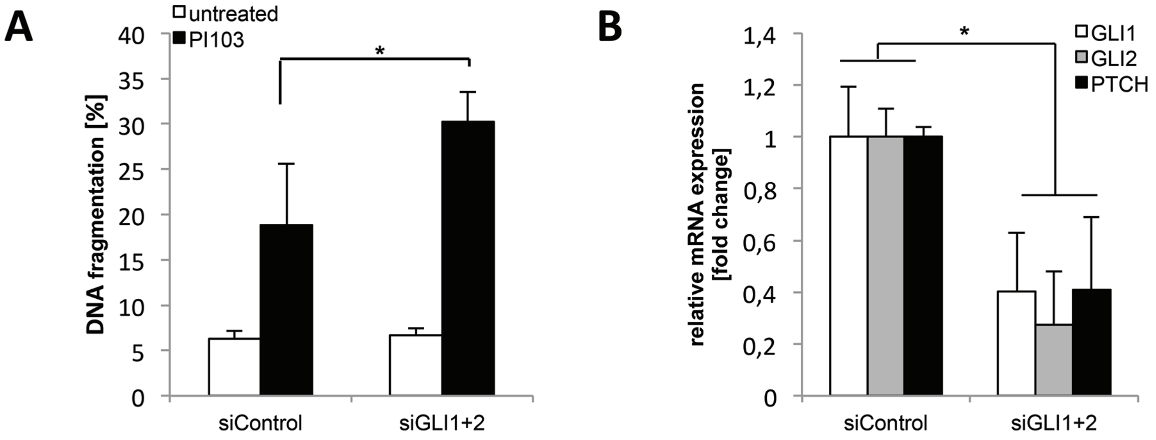

Supplementary Figure S3: Validation of HH inhibition via a genetic approach. (A) RD cells were transfected with siRNA targeting GLI1 and GLI2 and after 24 hours the cells were treated with 1 µM PI103. Apoptosis was determined by DNA fragmentation of PI-stained nuclei using flow cytometry. **(B)** the efficiency of GLI1 and GLI2 knockdown was determined by qRT-PCR 24 hours after transfection, as well as the target gene PTCH. Mean +/– S.D. of three independent experiments performed in triplicate are shown; *$p < 0.05$

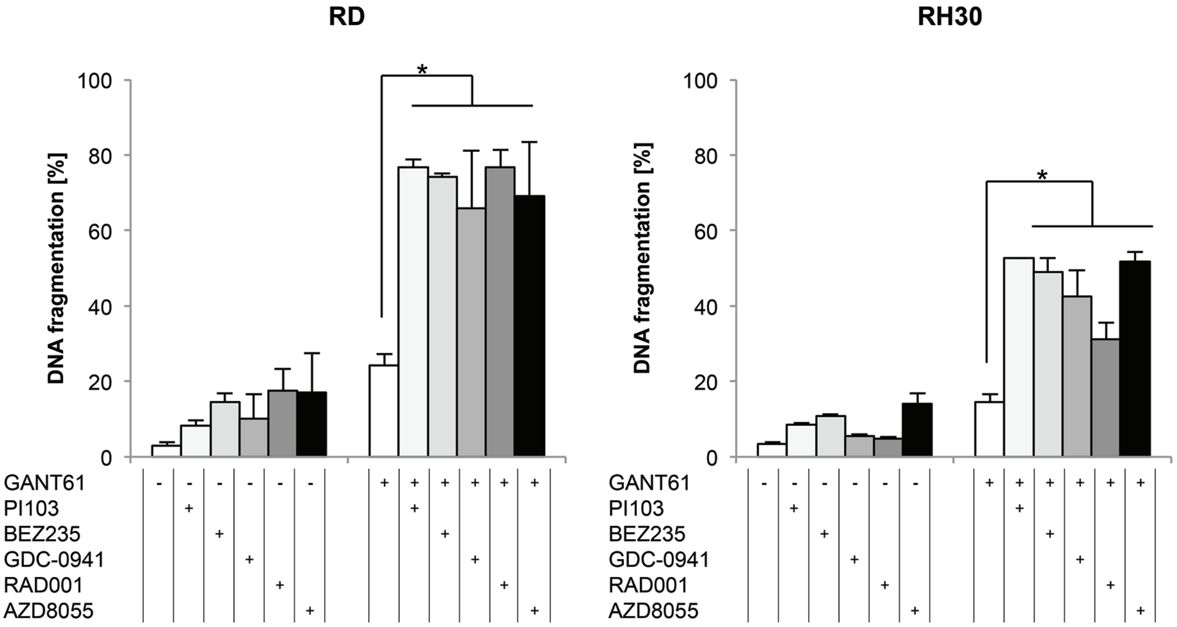

Supplementary Figure S4: Combinatory effect of PI3K and/or mTOR inhibition with HH inhibition. RD and RH30 cells were treated with 1 µM of indicated PI3K/AKT/mTOR inhibitors in combination with GANT61 (RD 6 µM; RH30 8 µM) and apoptosis was determined by DNA fragmentation of PI-stained nuclei using flow cytometry after 72 hours. Mean +/− S.D. of three independent experiments performed in triplicate are shown; *$p < 0.05$

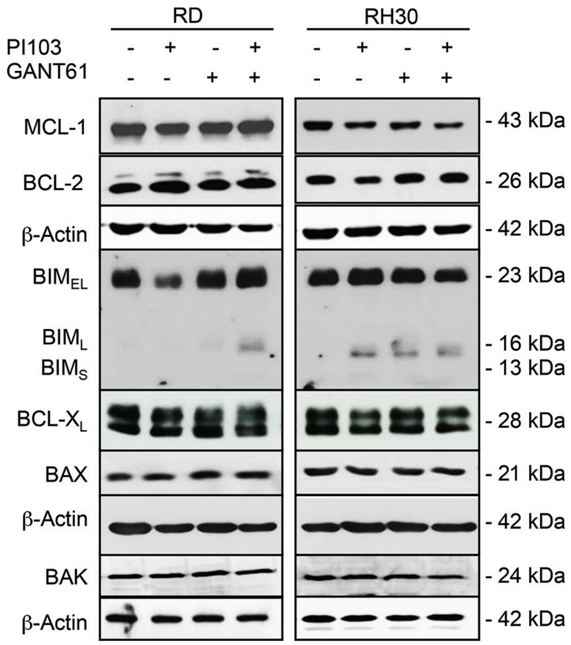

Supplementary Figure S5: Pro- and anti-apoptotic protein expression after GANT61/PI103 cotreatment. RD and RH30 cells were treated with 1 μM PI103 and/or GANT61 (RD 6 μM; RH30 8 μM) for 24 hours. Protein levels of pro- and anti-apoptotic proteins were determined by Western blotting. Representative blots of at least two different experiments are shown.

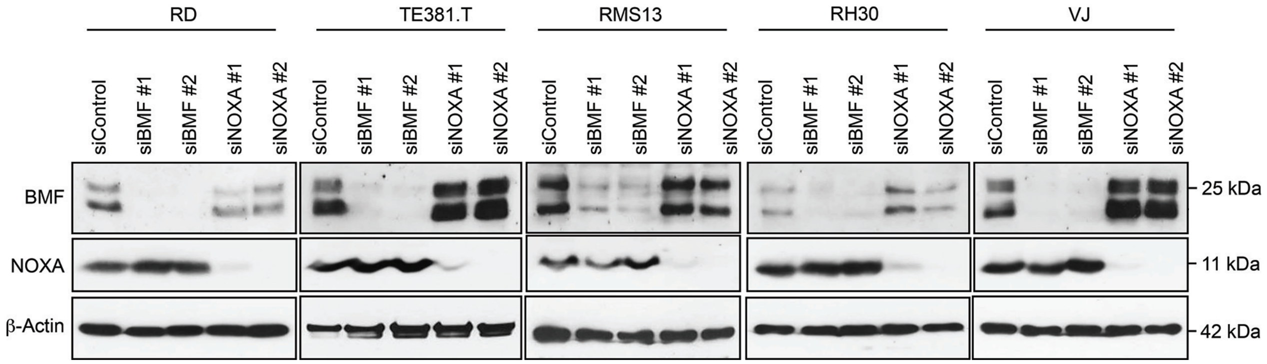

Supplementary Figure S6: Knockdown efficiency of BMF and NOXA. RD, TE381.T, RMS13, RH30 and VJ cells were transfected with siRNA against BMF or NOXA. After 24 hours cells were treated with 1 μM PI103 and GANT61 (RD 6 μM; TE381.T 8 μM; RMS13 10 μM; RH30 8 μM; VJ 8 μM) and proteins were isolated after 24 hours. Knockdown efficiency was determined by Western blotting. Representative blots of at least two different experiments are shown.

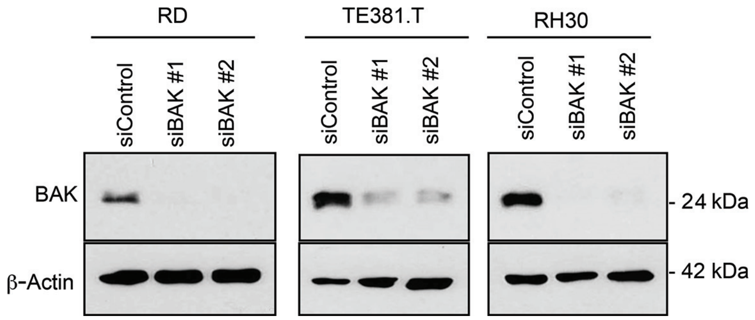

Supplementary Figure S7: Knockdown efficiency of BAK. RD, TE381.T and RH30 cells were transfected with siRNA targeting BAK and proteins were isolated after 24 hours. Knockdown efficiency was determined by Western blotting. Representative blots of at least two different experiments are shown.

Supplementary Table S1: Combination indices (CalcuSyn®). RMS cell lines were treated for 72 hours with indicated concentratione of PI103 and/or GANT61. Apoptosis was determined by FACS analysis of DNA fragmentation of propidium iodite-stained nuclei. Combination index (CI) was calculated by CalcuSyn software as described in Material and Methods for data shown in Fig. 1.

**RD**		**GANT61 [µM]**		
		**4**	**5**	**6**
PI103 [µM]	1	0.54	0.3	0.049
	1.5	0.494	0.294	0.106
	2	0.51	0.329	0.117

**RH30**		**GANT61 [µM]**		
		**4**	**6**	**8**
PI103 [µM]	1	0.503	0.493	0.54
	1.5	0.483	0.473	0.533
	2	0.397	0.468	0.524

**TE381.T**		**GANT61 [µM]**		
		**4**	**8**	**10**
PI103 [µM]	1	0.1247	0.2355	0.4253
	1.5	0.3262	0.512	0.69
	2	0.3484	0.6043	0.74

**VJ**		**GANT61 [µM]**		
		**2.5**	**5**	**10**
PI103 [µM]	0.5	0.799	0.624	0.325
	1	0.714	0.597	0.266
	2	0.586	0.427	0.271

**RMS13**		**GANT61 [µM]**		
		**8**	**10**	**12**
PI103 [µM]	1	0.612	0.687	0.814
	1.5	0.595	0.667	0.793
	2	0.608	0.678	0.795

Supplementary Table S2: siBMF and siNOXA combination vs. siBMF or siNOXA (students *t*-test)
RMS cells were treated as described in Fig. 4. Statistical analysis was performed using students *t*-test as described in Material and Methods comparing the double siRNA treated values with each comparing single treated one and *p*-values are listed.

RD	BMF #1 NOXA #1	BMF #1 NOXA #2	BMF #2 NOXA #1	BMF #2 NOXA #2
BMF #1	ns	$p \leq 0.05$	-	-
BMF#2	-	-	ns	ns
NOXA #1	ns	-	ns	-
NOXA #2	-	ns	-	ns

TE381.T	BMF #1 NOXA #1	BMF #1 NOXA #2	BMF #2 NOXA #1	BMF #2 NOXA #2
BMF #1	ns	ns	-	-
BMF#2	-	-	ns	ns
NOXA #1	ns	-	ns	-
NOXA #2	-	ns	-	ns

RMS13	BMF #1 NOXA #1	BMF #1 NOXA #2	BMF #2 NOXA #1	BMF #2 NOXA #2
BMF #1	ns	$p \leq 0.01$	-	-
BMF#2	-	-	ns	ns
NOXA #1	$p \leq 0.05$	-	$p \leq 0.05$	-
NOXA #2	-	$p \leq 0.05$	-	$p \leq 0.05$

RH30	BMF #1 NOXA #1	BMF #1 NOXA #2	BMF #2 NOXA #1	BMF #2 NOXA #2
BMF #1	ns	ns	-	-
BMF#2	-	-	ns	ns
NOXA #1	ns	-	ns	-
NOXA #2	-	ns	-	ns

VJ	BMF #1 NOXA #1	BMF #1 NOXA #2	BMF #2 NOXA #1	BMF #2 NOXA #2
BMF #1	ns	ns	-	-
BMF#2	-	-	ns	ns
NOXA #1	ns	-	ns	-
NOXA #2	-	ns	-	ns

Supplementary Table S3: Primer Sequences (RT-PCR)

Primer	Sequence
h_28S_for	TTGAAAATCCGGGGGAGAG
h_28S_rev	ACATTGTTCCAACATGCCAG
h_Gli1_for	AGCTACATCAACTCCGGCCA
h_Gli1_rev	GCTGCGGCGTTCAAGAGA
h_Gli2_for	AGCAAGGTCAAGACCGAGCCT
h_Gli2_rev	TCTCTTGGTGCAGCCTGGGAT
h_Gli3_for	ACCATTACGATCCATCTCCGA
h_Gli3_rev	TAAGTGACCATAGGAGCCACT
h_Ptch_for	GAGGTTGGTCATGGTTACATGGA
h_Ptch_rev	TGCTGTTCTTGACTGTGCCACC
h_Shh_for	GATGACTCAGAGGTGTAAGGAC
h_Shh_rev	CCTCGTAGTGCAGAGACTCC
h_Noxa_for	GGAGATGCCTGGGAAGAAG
h_Noxa_rev	CCTGAGTTGAGTAGCACACTCG
h_Bmf_for	GAGACTCTCTCCTGGAGTCACC
h_Bmf_rev	CTGGTTGGAACACATCATCCT
h_BIM_for	CATCGCGGTATTCGGTTC
h_BIM_rev	GCTTTGCCATTTGGTCTTTTT
h_Puma_for	GACCTCAACGCACAGTACGA
h_Puma_rev	GAGATTGTACAGGACCCTCCA
